# Proteinaceous Effector Discovery and Characterization in Plant Pathogenic *Colletotrichum* Fungi

**DOI:** 10.3389/fmicb.2022.914035

**Published:** 2022-05-27

**Authors:** Xinyu Lu, Jinlu Miao, Danyu Shen, Daolong Dou

**Affiliations:** Department of Plant Pathology, Nanjing Agricultural University, Nanjing, China

**Keywords:** *Colletotrichum*, effector, prediction, function, pathogen-plant interaction

## Abstract

Anthracnose caused by plant pathogenic *Colletotrichum* fungi results in large economic losses in field crop production worldwide. To aid the establishment of plant host infection, *Colletotrichum* pathogens secrete numerous effector proteins either in apoplastic space or inside of host cells for effective colonization. Understanding these effector repertoires is critical for developing new strategies for resistance breeding and disease management. With the advance of genomics and bioinformatics tools, a large repertoire of putative effectors has been identified in *Colletotrichum* genomes, and the biological functions and molecular mechanisms of some studied effectors have been summarized. Here, we review recent advances in genomic identification, understanding of evolutional characteristics, transcriptional profiling, and functional characterization of *Colletotrichum* effectors. We also offer a perspective on future research.

## Introduction

Anthracnose, which is caused by the fungal genus *Colletotrichum*, is one of the most devastating agricultural diseases (Stephenson et al., 2000; Yoshino et al., 2012; Chen et al., 2021). Over 600 species of *Colletotrichum* have been identified and classified as singletons or species complexes (Irieda et al., 2014). They can infect a large variety of plants worldwide including vegetables, fruit plants, forest trees, cereals, and legumes (O’Connell et al., 2012; Gan et al., 2013; Irieda et al., 2014). *Colletotrichum graminicola* causes anthracnose leaf blight and stalk rot of maize and sorghum, respectively (Sanz-Martin et al., 2016). *C. lentis* is the causative agent of anthracnose on soybean, lentil, and pea. *C. higginsianum* mainly infects Brassicaceae plants (Huser et al., 2009), and *C. orbiculare* prefers to attack cucurbitaceous plants (Perfect et al., 1999). Moreover, *C. higginsianum* and *C. orbiculare* can also infect the model plants *Arabidopsis thaliana* and *Nicotiana benthamiana*, respectively, providing pathosystems for studies of pathogen–plant interactions (O’Connell et al., 2004). Because of its extreme destruction, widespread distribution, and scientific importance as a model pathogen–plant interaction system, the genus *Colletotrichum* has been ranked among the top 10 most important phytopathogenic fungi in the world (Dean et al., 2012). Presently, the two main strategies to control anthracnose are breeding resistant sources and using chemical fungicides. However, the complex genetic variation of *Colletotrichum* strains leads to a loss of cultivar resistance and emergence of fungicide resistance, which makes anthracnose difficult to control. Therefore, to devise strategies to efficiently control the spread of the disease, it is urgent to thoroughly clarify the molecular mechanism of *Colletotrichum* pathogenicity.

*Colletotrichum* fungi can infect multiple plant parts such as leaves, stems, and fruits. Upon contact with the plant, *Colletotrichum* conidium initially adheres to the host surface, and germinates to form germ tubes. Then, a specialized infection structure called the melanized appressorium forms at the tip of the germ tube and penetrates the host (Kleemann et al., 2012; Irieda et al., 2014). Post-penetration, the majority of *Colletotrichum* pathogens adapt a hemibiotrophic lifestyle. They develop penetration peg at the infection point to invade plant cells, and then produce specialized infection structures such as bulbous vesicles and primary hyphae to obtain nutrients from living plant tissues. *Colletotrichum* fungi switch to a necrotrophic stage, after which they produce secondary hyphae that invade neighboring cells and kill host tissues (Perfect et al., 1999).

To establish successful infection, phytopathogenic fungi typically secrete a large number of virulent effectors into host cells (Dou and Zhou, 2012). Effectors are proteins secreted by pathogens to manipulate plant physiology and immunity, to facilitate infection, trigger plant defense responses or both (Bozkurt et al., 2012; Rafiqi et al., 2012). According to their subcellular localization, fungal effectors can be classified into two major groups: apoplastic effectors act in extracellular spaces while intracellular ones function inside host cells (Dou and Zhou, 2012). Generally, fungal effector proteins contain an N-terminal signal peptide and are secreted via the conventional endoplasmic reticulum–Golgi apparatus secretion pathway (Giraldo et al., 2013). These fungal effectors typically lack sequence similarity to known proteins, which is thought to be the result of the evolutionary pressure that promotes the rapid diversification of effector activities, to avoid recognition by the plant immune system. Also, many effectors are small cysteine-rich proteins containing unidentified motifs and domains. Evolutional and functional studies of effectors have been important for comprehensive understanding of plant–pathogen interactions, and have facilitated the development of more effective and eco-friendly approaches to disease control.

Recent advances in high-throughput sequencing have yielded over 50 genomes of *Colletotrichum* pathogens. Computational predictions suggest that there are genes for hundreds of putative effectors in these genomes (O’Connell et al., 2012; Gan et al., 2013; Buiate et al., 2017; Lelwala et al., 2019). Comparative studies reveal that each *Colletotrichum* species contains both conserved and unique effectors, which are likely to play crucial roles in their adaptation to plant hosts. Furthermore, functional investigations of some *Colletotrichum* effectors suggest a clear contribution to the pathogenic success. Effectors have been the main focus of research on the interaction between *Colletotrichum* pathogens and plants because they directly affect the invasion, expansion, and disease occurrence of *Colletotrichum* pathogens. In this review, we summarize recent advances toward the identification and functional characterization of putative effectors from *Colletotrichum* pathogens. We focus on the genomic identification, evolutionary characteristics, and transcriptional profiling of effectors, and then on recent progress in elucidating their biological functions and effects on compatibility.

## Genomic Identification of Candidate Effectors

**FIGURE 1:**
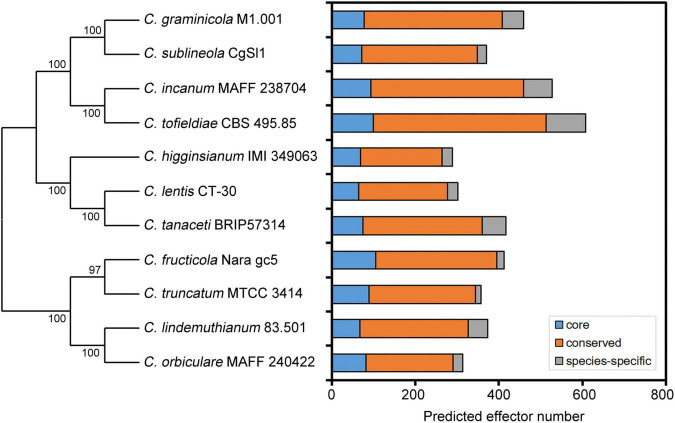
Conservation patterns of putative effector proteins from *Colletotrichum* pathogens. A neighbor-joining species phylogeny was drawn based on the alignment of single-copy orthologs. Bootstrap values are based on 1,000 replicates. The effector candidates were predicted using a streamlined bioinformatics analysis in this study. Core: core effectors which were present in each *Colletotrichum* species. Conserved: conserved effectors which had orthologs in other *Colletotrichum* species. Species-specific: species-specific effectors.

With the rapid development of high-throughput sequencing technologies and bioinformatics tools, analysis of entire genomes has become common practice, and can establish causal relationships between genome characteristics and the biology of plant pathogens (Raffaele and Kamoun, 2012; Plissonneau et al., 2017). So far, at least 50 genomes of *Colletotrichum* pathogens have been fully sequenced. Prediction of candidate effectors is the first step in the functional investigation of these proteins. Because fungal effectors do not possess typical motifs or other conserved sequence features, their prediction *in silico* is challenging. Note that the definition of effector proteins varies considerably among authors. Generally, computational prediction methods first predict secreted proteins, and then apply the EffectorP prediction tool, which screens high-priority effector candidates based on sequence length, molecular weight and protein net charge, as well as cysteine, serine and tryptophan content (Buiate et al., 2017; Lelwala et al., 2019). Some studies also define small secreted proteins (SSPs) as putative effectors; these are typically cysteine-rich and less than 300 amino acids in length (O’Connell et al., 2012; Gan et al., 2013). It may be important for phytopathogens to maintain effector proteins with a relatively small molecular weight for easier secretion. In recent years, several novel prediction strategies have been developed by including additional features, such as secretome analysis and *in planta* expression based on transcriptomic analysis (Ashwin et al., 2017). Due to criterion variation among studies related to effector definition and prediction, it is biased to investigate the effector size variation among *Colletotrichum* pathogens. Therefore, we predicted putative effector proteins in each *Colletotrichum* genome using a streamlined bioinformatics analysis. Secretome was predicted using a series of tools. SignalP 5.0 and WoLF-PSORT were performed to identify signal peptides and extracellular localization, respectively. TMHMM v2.0 and PredGPI were used to exclude sequences with transmembrane helices and GPI anchors, respectively. Sequences were then submitted to EffectorP 3.0 for effector prediction. Analyses of genomes have uncovered large inventories of candidate effectors (288–608 per genome) in different *Colletotrichum* species (Figure 1 and Table 1), suggesting that putative effector numbers vary considerably among *Colletotrichum* species. Further cluster analysis in the genus *Colletotrichum* identified ∼20% of core effectors which were present in each *Colletotrichum* species, while another 70% of conserved effectors had orthologs in other *Colletotrichum* species. Moreover, each *Colletotrichum* species contained 4.1–15.6% of species-specific effectors (Figure 1). These data suggested that the conservation patterns of candidate effectors appear to be related to the host range and virulence of *Colletotrichum* pathogens.

**TABLE 1:** List of genome assembly and predicted candidate effector information in *Colletotrichum* pathogens.

Species	Strain	Host	Genome size (Mb)	Total gene number	Putative effector number^#^	Accession number	References
*C. graminicola*	M1.001	*Zea mays*	50.9	12,006	314	ACOD01000001	O’Connell et al., 2012
*C. higginsianum*	IMI 349063	Brassicaceae	49.3	16,172	417	CACQ00000000.2	O’Connell et al., 2012
*C. orbiculare*	MAFF 240422	*cucurbits*	88.3	13,479	459	AMCV00000000.2	Gan et al., 2013
*C. fructicola* (deposited as *C. gloeosporioides*)	Nara gc5	Fruits	55.6	15,469	608	ANPB00000000.1	Gan et al., 2013
*C. tofieldiae*	CBS 495.85	*Tofieldia calyculata*	53.5	13,425	412	LFHP00000000.1	Hacquard et al., 2016
*C. incanum*	MAFF 238704	Brassicaceae, fabaceae, and solanaceae	53.6	13,665	357	LFIW00000000.1	Hacquard et al., 2016
*C. sublineola*	CgSl1	sorghum	64.8	13,311	373	MQVQ01000001	Buiate et al., 2017
*C. truncatum*	MTCC 3414	*Capsicum annuum*	55.4	13,724	528	NBAU00000000	Rao et al., 2018
*C. tanaceti*	BRIP57314	*Tanacetum cinerariifolium*	57.9	12,172	288	PJEX00000000.1	Lelwala et al., 2019
*C. lentis*	CT-30	Legume	56.1	11,436	301	NWBT00000000.1	Bhadauria et al., 2019
*C. lindemuthianum*	83.501	*Phaseolus vulgaris*	97.4	11,673	370	MASO00000000.2	de Queiroz et al., 2019

*^#^Due to criterion variation among studies related to effector definition and prediction, we predicted putative effector proteins using a streamlined bioinformatics analysis in this study.*

## Evolutionary Characteristics of Effectors

Through comparative genomics analysis, the large number of *Colletotrichum* effectors could be divided into two classes based on their conservation patterns: lineage-specific effector candidates, which have no homology to any other protein (species-specific) or have homolog only to proteins from the same genus (genus-specific); and conserved effectors, which have homology to proteins from other fungal genera (O’Connell et al., 2012; Baroncelli et al., 2016). Compared to *C. graminicola*, *C. higginsianum* has twice as many as candidate effectors; moreover, 72% of them are species-specific, suggesting that the more diversified effector repertoire of *C. higginsianum* might be related to its broader range of host plants (O’Connell et al., 2012). In another case, *C. graminicola* and *C. sublineola* share a close evolutionary relationship, but are completely host-specific. Comparative genomic studies indicated that 32 candidate effectors are specific to *C. graminicola*, and 21 appear to be specific to *C. sublineola*; these may participate in early events related to host recognition (Buiate et al., 2017). Three strains of *C. scovillei*, Coll-153, Coll-524, and Coll-365, exhibit variable virulence in chili pepper. Comparative genomic analysis showed that the strain Coll-524 has a remarkably greater number of candidate effectors than Coll-153 and Coll-365, and these varied effectors are mainly found in the acutatum complex (Hsieh et al., 2022). The large number of effectors may contribute to the high virulence of Coll-524. In *C. tanaceti*, a minority of the effectors share similarity with those of other tested *Colletotrichum* species, which emphasizes their role in adaptation to new hosts (Lelwala et al., 2019). As well as lineage-specific effectors, conserved effectors have also been studied because they are typically important for infection of a wide range of plants. Comparison of the repertoire of candidate effectors among four *Colletotrichum* species pathogenic to soybean showed that 84% of *C. musicola*, 85% of *C. sojae*, 80% of *C. truncatum*, and 83% of *C. musicola* effectors are conserved not only within the *Colletotrichum* genus, but also in other microorganisms (Boufleur et al., 2021). Thus, the conservation patterns of candidate effectors appear to be related to the host range and virulence of *Colletotrichum* pathogens.

Of the large repertoire of predicted candidate effectors in *Colletotrichum* genomes, studies have found that the majority are small cysteine-rich proteins. In C. *higginsianum* and *C. graminicola*, the predicted candidate effectors are mostly small secreted proteins (SSPs), with typical lengths ranging between 110 and 175 residues, and are more cysteine-rich than the total proteome (O’Connell et al., 2012). Of the predicted SSPs, 49.4% in *C. fructicola* and 54.6% in *C. orbiculare* are cysteine-rich proteins (Gan et al., 2013). Similarly, such characteristics have also been found in putative effectors derived from *C. sublineola*, *C. truncatum*, *C. tanaceti*, and *C. lentis* (Buiate et al., 2017; Bhadauria et al., 2019; Lelwala et al., 2019).

Notably, many candidate effectors are homologs of known effectors from other phytopathogens, such as biotrophy-associated secreted (BAS) protein 2 from *Magnaporthe oryzae* (Mosquera et al., 2009), Ecp6 from *Cladosporium fulvum* (de Jonge et al., 2010), necrosis and ethylene-inducing protein 1 (Nep1)-like proteins (NLPs) from *Phytophthora* pathogens, MC69 from *M. oryzae* (Saitoh et al., 2012), and secreted in xylem (SIX) 5 protein from *Fusarium oxysporum* (Lievens et al., 2009). Furthermore, some *Colletotrichum* effector proteins contain functional domains. According to an analysis of the Pfam database, 46, 21, and 75 putative effector proteins in *C. sublineola*, *C. graminicola*, and *C. truncatum*, respectively, have functional domains (Buiate et al., 2017; Rao and Nandineni, 2017). Several domains, such as CFEM domain, chitin-binding domain, lysin motif (LysM) domain, and NPP1 domain, are related to pathogenesis. The CFEM domain is composed of eight conserved cysteine residues, and proteins containing this domain are important in pathogenesis (DeZwaan et al., 1999). *C. graminicola* M1.001 and *S. sublineola* CgSl1 share 10 and 11 SSPs, respectively, containing the CFEM domain (Buiate et al., 2017). Both of these species also contain two SSPs containing the chitin-binding domain, which is thought to bind to the chitin present in fungal cell walls to protect the pathogen from plant chitinases (van Esse et al., 2007). NLPs containing the NPP1 domain form a large and conserved family in filamentous pathogens, and members have a strong ability to induce necrosis in dicot plants (Pemberton and Salmond, 2004). The NPP1 domain has been identified in putative effectors of many *Colletotrichum* species such as *C. higginsianum*, *C. sublineola*, and *C. graminicola* (O’Connell et al., 2012; Buiate et al., 2017).

Many genomes of *Colletotrichum* pathogens, such as *C. higginsianum* and *C. gloeosporioides*, contain both core chromosomes and minichromosomes (Plaumann et al., 2018). Supernumerary minichromosomes are common in this genus. The minichromosomes display low gene density, are highly enriched in transposable elements (TEs), and are shown to be virulence determinants on host plants. Analysis of the genomes of the strawberry-pathogenic *C. fructicola*, *C. siamense*, and *C. aenigma* strains identified effector gene clusters in the repeat-rich minichromosomes (Gan et al., 2021). In *C. higginsianum*, the minichromosomes are more enriched with putative effectors than the core genome, including seven that are strongly induced during infection (Dallery et al., 2017; Tsushima et al., 2019). Furthermore, analysis of the *C. tanaceti* genome show that the genomic distances between TEs and effector genes are smaller than that between TEs and random genes, suggesting that TEs are close to putative effector genes (Lelwala et al., 2019). Similarly, a significant association was detected between TEs and genes encoding putative effectors in *C. higginsianum* and *C. truncatum* (Dallery et al., 2017; Rao et al., 2018). These observations suggest that repeat-rich genomic regions tend to harbor genes that encode putative effectors and evolve at higher rates.

In the arms race model of evolution, phytopathogen pathogenicity proteins commonly evolve faster to avoid host recognition. Consistently, the analysis of selective pressure of all of the protein-coding sequences in *C. graminicola* showed that 224 genes undergo positive selection; such genes mainly code for putative effectors and other putative virulence factors (Rech et al., 2014). This evidence for positive selection of these putative effectors suggests that they likely evolve rapidly in response to different ecological niches.

## Transcriptional Profiling of Candidate Effectors

Many *Colletotrichum* pathogens employ a hemibiotrophic strategy and express effectors at different stages of the infection process, including before penetration of the interface, after appressorium penetration, and during the biotrophic and necrotrophic stages. Therefore, RNA sequencing technology has been widely applied to *Colletotrichum* pathogens such as *C. graminicola*, *C. higginsianum*, and *C. fructicola* at different development and infection stages. The availability of transcriptomes enables analysis of the transcriptional profiling of *Colletotrichum* candidate effectors at different stages of hemibiotrophic infection, supporting investigation of the infection phase-specific virulence roles of effectors (O’Connell et al., 2012; Bhadauria et al., 2017; Liang et al., 2018). In a microarray study of *C. orbiculare* gene expression regulation during infection of *N. benthamiana*, many SSPs were upregulated in the initial colonization stage (Gan et al., 2013). Deep transcriptome sequencing of *C. higginsianum* associated with different infection stages yielded 198 unigenes encoding candidate effectors, of which 102 are not expressed in the late necrotrophic phase. Thus, these genes are considered biotrophy-associated candidate effectors, which are relevant to appressorium penetration and the development of biotrophic hyphae (Kleemann et al., 2012). Genome-wide expression profiling of *C. graminicola* and *C. higginsianum* genes showed that most effectors are strongly induced during biotrophy. Intriguingly, one gene (*ChEC6*) encoding a candidate effector is the most strongly induced by host contact, and its transcription begins in the appressorium and continues in young biotrophic hyphae (O’Connell et al., 2012). Another set of transcriptomic data associated with *C. fructicola*–strawberry interactions revealed that 15 of the top 100 upregulated *C. fructicola* genes encode candidate effectors during plant invasion, which is the first step of infection (Zhang et al., 2018). These findings suggest that candidate effectors with transcriptional induction at an early stage of the infection process may function in host defense suppression. Also, analysis of gene expression in *C. gloeosporioides* during necrotrophy revealed that 149 SSPs are specifically expressed at that stage. Among them, a necrotrophic-stage specific SSP encoding NLP is highly upregulated, underlining the need for rapid host cell killing (Alkan et al., 2015).

In addition to analysis based on RNA sequencing date, the transcriptional patterns of some effectors with known virulence functions have also been studied deeply. For example, *CgDN3* from *C. gloeosporioides* encodes a determinant of pathogenicity associated with the biotrophic phase to regulate hyphal extension, and its homologue CoDN3 suppresses the hypersensitive reaction (HR)-like response triggered by necrosis-inducing proteins (Stephenson et al., 2000; Yoshino et al., 2012; Isozumi et al., 2019). Reverse transcriptase polymerase chain reaction analysis showed that *CgDN3* is expressed during the biotrophic stage 1-4 days after inoculation (Stephenson et al., 2000). Another two effectors, ChELP1 and ChELP2, of *C. higginsianum* prevent host chitin recognition in immune responses by associating with chitin polymer and oligomers. Expression patterns of *ChELP* genes revealed that *ChELP1* and *ChELP2* are the most expressed among them, and are strongly induced during the early biotrophic phase (Takahara et al., 2016). NLPs are widely distributed across many pathogenic fungi. In contrast with effectors expressed during the early phases of infection, the *NLP* genes of *C. orbiculare* and *C. higginsianum* are expressed specifically at the onset of necrotrophic growth and have the potential to cause necrotic lesions and accelerate host death (Kleemann et al., 2012; Azmi et al., 2018; Chen et al., 2021). These results indicate that *Colletotrichum* effectors are host-induced and expressed in consecutive waves associated with the hemibiotrophic infection mode. Most effectors expressed during initial host penetration and biotrophic phase either act on appressorium-mediated penetration or host defense suppression, whereas others expressed precisely at the onset of necrotrophic growth can induce cell death to accelerate the switch to that stage of infection. Remarkably, some *C. higginsianum* effectors such as ChEC3 and ChEC3a, are induced at biotrophic phase, and also suppress cell death induced by ChNLP1, suggesting that *C. higginsianum* effectors interfere with ChNLP1-specific signaling components and thereby maintain host viability during initial biotrophic growth (Kleemann et al., 2012).

## Effectors for Infection Structure Formation

During the infection process, some *Colletotrichum* effectors may play a role in pathogenicity by regulating the development of the infection structures. For example, two LysM proteins (ChELP1 and ChELP2) in *C. higginsianum* have been shown to affect appressorium-mediated penetration. ChELP2 preferentially accumulates on the surface of biotrophic primary hyphae but is absent on necrotrophic secondary hyphae. ChELP1 RNAi mutants show considerably more abnormal spore germination than wild-type ones, and produce appressorium that fail to penetrate plant epidermal cells (Takahara et al., 2016). These data suggest that ChELP1 and ChELP2 execute their virulence functions in a penetration ability-dependent manner. Similarly, *C. gloeosporioides* genome encodes a CgDN3 effector, which was identified as a virulence effector by its induction under conditions of nitrogen deprivation. CgDN3 mutants replace the *CgDN3* gene with a chimeric hygromycin resistance gene, which causes a severe reduction in the rate of appressorium formation in vitro and aids fungal infection (Stephenson et al., 2000). Taken together, the *Colletotrichum* effectors control infection structures to potentially induce pathogenicity.

## Effector Delivery and Subcellular Localization

Effector delivery is associated with the hemibiotrophic lifestyle of *Colletotrichum* pathogens. In *C. higginsianum*, the cytological analysis shows that effectors localize to stage-specific compartments at the host-pathogen interface (Kleemann et al., 2012). Some early-expressed effectors such as ChEC36 and ChEC6 specifically localize to the penetration pore, suggesting that they are focally secreted from appressorial penetration pores before host invasion. In addition, some later-expressed effectors including ChEC89, ChEC3, ChEC13, and ChEC34, accumulate in interfacial bodies on the surface of biotrophic hyphae, implicating these hyphae in effector delivery (Kleemann et al., 2012). In another study, *C. orbiculare* effectors exhibit ring-shaped accumulations around the neck of the biotrophic hyphae (Irieda et al., 2014).

Once pathogens deliver effectors into plants, it is critical to identify host cell compartment that effectors target and how they function. Many fungal and oomycete effectors have been reported to target diverse plant compartments, such as nuclei, vacuole, tonoplast, plasma membrane, and cytosol (Petre et al., 2015). Of the 61 biotrophy-expressed effector in *C. higginsianum*, nine are specifically imported into plant nuclei (Robin et al., 2018), suggesting that manipulation of plant nuclear processes is an important virulence strategy of *Colletotrichum* pathogens. Meanwhile, seven others label plant Golgi, peroxisomes and microtubules, which are not previously reported (Robin et al., 2018). Understanding the subcellular localization of *Colletotrichum* effectors can give valuable insights into plant proteins that they potentially interact with, and how they manipulate plant processes.

## Effectors Block Fungal Chitin-Induced Host Signaling

Chitin is an important component in the cell walls of all pathogenic fungi, and acts as a microbe-associated molecular pattern (MAMP) that can be recognized by plant chitin receptors to activate a variety of MAMP-triggered immune responses (Miya et al., 2007; Dodds and Rathjen, 2010). Almost all of the chitin receptors have been identified as LysM-containing proteins, and three LysM domains are essential for chitin binding (Tanaka et al., 2013). To evade recognition by host chitin receptors, pathogenic fungi secrete effectors to compete with them (to bind to and protect chitin against recognition), or act as plant chitinase-degrading enzymes to specifically degrade chitinase and suppress the release of chitin fragments directly captured by plant cells. The Brassicaceae anthracnose fungus *C. higginsianum* encodes many LysM-containing proteins. Among these, ChELP1 and ChELP2 have a signal peptide and accumulate in an early biotrophic phase. The recombinant proteins of ChELP1 and ChELP2 show high affinity for chitin fragments in vitro and specifically bind fungal cell wall chitin. Analysis of mitogen-activated protein kinase (MAPK) activation showed that both ChELP1 and ChELP2 can suppress this chitin-triggered MAPK activation by sequestering chitin (Takahara et al., 2016). Another LysM-containing protein, Cgfl, which is a conserved fungalysin metalloprotease in *C. graminicola*, can bind plant chitinase for degradation. Δ*Cgfl* mutants reduce this ability in colonized maize leaves and roots. Also, the culture filtrates of Δ*Cgfl* show significantly reduced proteolytic activity in response to the substrate casein *in vitro*. Inoculation of maize leaves with Δ*Cgfl* increases plant chitinase activity compared with inoculation with the *C. graminicola* wild-type strain (Sanz-Martin et al., 2016). These results suggest that Cgfl targets degradation of plant chitinase to protect chitin released from pathogens against cleavage and recognition. As well as chitin signaling, MAPK and plant hormone signaling including salicylic acid, jasmonic acid, and ethylene, participate in defense responses. Although the evidence shows that effectors play a role in suppressing chitin signaling and chitin-induced immunity, no *Colletotrichum* effectors have shown the ability to target and interfere with plant hormone signaling yet.

## Effectors Suppress Hypersensitive Cell Death

Plants have evolved a sophisticated innate immune system, and the extracellular (apoplastic) and intracellular (cytoplasmic) spaces are major sites of pathogen molecule recognition and plant defense. Pathogen-associated molecular pattern (PAMP)-triggered immunity (PTI) is a substantial barrier in the apoplastic space of plants, and impedes microbial infection (Wang and Wang, 2018). Plant pattern-recognition receptors (PRRs) that recognize PAMP associate with membrane-localized receptor-like kinases (RLKs) and receptor-like cytoplasmic kinases (RLCKs) to transduce defense signaling and trigger immune responses (Monaghan and Zipfel, 2012). There is growing evidence that Brassinosteroid insensitive 1-associated kinase1 (BAK1) is an important RLK that participates in different signaling pathways to modulate various types of programmed cell death (He et al., 2007; Kemmerling et al., 2007; Jeong et al., 2010). Accordingly, pathogens have developed effectors to suppress cell death as a defense mechanism and promote pathogen infection.

The cucumber anthracnose fungus *C. orbiculare* secretes necrosis-inducing secreted protein 1 (NIS1), which is a conserved effector in filamentous fungal that targets host core immune components. The homolog CoNIS1 can suppress the cell death induced by the oomycete PAMP INF1 by interacting with BAK1 to inhibit its kinase activity. The *Arabidopsis bak1-5* mutant encodes a semidominant allele of BAK1. Inoculation of *C. higginsianum* onto the *bak1-5* mutant facilitates pathogen infection compared to inoculation onto Col-0. This finding suggests that *Colletotrichum* pathogens deploy a core effector to attacks the conserved immune component (Irieda et al., 2019). Interestingly, phylogenetic analysis reveals that NIS1 is widely conserved in fungal pathogens. ChNIS1 of the crucifer anthracnose fungus *C. higginsianum* and MoNIS1 of *M. oryzae* can also suppress INF1-induced cell death in *N. benthamiana* (Irieda et al., 2019). Similarly, two virulence-related effectors of *C. orbiculare*, SIB1 and SIB2, can suppress the cell death response triggered by INF1. Overexpression of *SIB1* and *SIB2* increases the susceptibility of *N. benthamiana* to *C. orbiculare* (Zhang et al., 2021). Like the oomycete PAMP INF1, a mammalian proapoptotic molecule, BAX, induces programmed cell death (PCD), which is similar to the defense-related HR. Therefore, it typically acts as a reference for the HR-suppressing ability of pathogen effectors (Lacomme and Santa Cruz, 1999; Dou et al., 2008). The *C. fructicola* effector CfEC92 is an important virulence factor that infects both apple leaves and fruits. Sequence similarity analysis of CfEC92 homologs showed that this SSP is conserved across the genus *Colletotrichum*. Overexpressing *CfEC92* can suppress BAX-induced cell death in *N. benthamiana* (Shang et al., 2020). These data suggest that CfEC92 and its homolog proteins possess a cell death-suppressing function for the host immune response.

Necrosis and ethylene-inducing protein 1-like proteins and NIS1 have an extremely broad distribution in filamentous fungi (Yoshino et al., 2012; Seidl and Van den Ackerveken, 2019). Filamentous phytopathogens have evolved effector proteins that can suppress the host cell death triggered by NLPs and NIS1 to promote disease. *C. higginsianum* effector candidates (ChECs) are co-expressed with the cell death-inducing protein ChNLP1 in *N. benthamiana* and exhibit virulence. Among the 102 ChECs, ChEC3, ChEC3a, ChEC5, ChEC6, and CHEC34 have been screened and exhibit significant cell death-suppressing activity. Western blot analysis indicates that these ChECs have no impact on ChNLP protein stability, and therefore reduce the necrosis of ChNLP in a suppression activity-dependent manner (Kleemann et al., 2012). As a further example, both the effector CgDN3 of *C. gloeosporioides* and its homolog in *C. orbiculare*, as pathogenicity-related proteins, suppress the HR-like response induced by NIS1 (Stephenson et al., 2000; Yoshino et al., 2012). Subsequently, sequence analysis has shown that CoDN3 suppresses the necrotic lesions caused by NLP1 homologues in a CaM-binding domain-dependent manner (Isozumi et al., 2019). Taken together, these data illustrate that a conserved strategy is employed by *Colletotrichum* species, involving the deployment of virulence effectors to suppress cell death and therefore counter plant immunity (Table 2).

**TABLE 2:** List of plant immunity-suppressing effectors in *Colletotrichum* pathogens.

Effector	*Colletotrichum* pathogen	Expression stage	Biological functions	Sequence conservation	References
ChELP1	*C. higginsianum*	Biotrophic phase	Binging chitin polymer and oligomers, suppressing chitin-triggered plant immune responses, and contributing to fungal virulence and appressorium-mediated penetration	Conserved in fungi	Takahara et al., 2016
ChELP2	*C. higginsianum*	Biotrophic phase	Binging chitin polymer and oligomers, suppressing chitin-triggered plant immune responses, and contributing to fungal virulence and appressorium-mediated penetration	Conserved in fungi	Takahara et al., 2016
ChNIS1	*C. higginsianum*	Unknown	Suppressing INF1-induced cell death and PAMP-triggered ROS generation	Conserved in fungi	Irieda et al., 2019
ChEC3	*C. higginsianum*	Biotrophic phase	Suppressing cell death	Conserved in *Colletotrichum* spp.	Kleemann et al., 2012
ChEC3a	*C. higginsianum*	Biotrophic phase	Suppressing cell death	Conserved in *Colletotrichum* spp.	Kleemann et al., 2012
ChEC5	*C. higginsianum*	Saprotrophic mycelium	Suppressing cell death	Conserved in fungi	Kleemann et al., 2012
ChEC6	*C. higginsianum*	Biotrophic phase	Suppressing cell death	Unknown	Kleemann et al., 2012
CHEC34	*C. higginsianum*	Biotrophic phase	Suppressing cell death	Unknown	Kleemann et al., 2012
CoNIS1	*C. orbiculare*	Unknown	Suppressing INF1-induced cell death, inhibiting ROS generation triggered by flg22 and chitin, and interacting with BAK1 and BIK1 to inhibit their kinase activities	Conserved in fungi	Irieda et al., 2019
SIB1	*C. orbiculare*	Early infection stage	Suppressing *N. benthamiana* immunity, inhibiting INF1-induced cell death, and suppressing ROS generation triggered by flg22 and chitin	Conserved in *Colletotrichum* spp.	Zhang et al., 2021
SIB2	*C. orbiculare*	Unknown	Suppressing *N. benthamiana* immunity	Conserved in *Colletotrichum* spp.	Zhang et al., 2021
CoDN3	*C. orbiculare*	Biotrophic phase	Suppressing necrotic lesion of NIS1 and NLP1	Conserved in *Colletotrichum* spp.	Yoshino et al., 2012; Isozumi et al., 2019
Cgfl	*C. graminicola*	Biotrophic phase	Contributing to pathogenicity, degrading chitinases produced by plants	Conserved in fungi	Sanz-Martin et al., 2016
CfEC92	*C. fructicola*	Early infection stage	Contributing to pathogenicity, suppressing BAX-triggered cell death, and inhibiting a subset of plant defense-related gene expression	Conserved in *Colletotrichum* spp.	Shang et al., 2020
CgDN3	*C. gloeosporioides*	Biotrophic phase	Contributing to pathogenicity, maintaining appressoria formation, and suppressing cell death induced by NIS1	Conserved in *Colletotrichum* spp.	Stephenson et al., 2000; Yoshino et al., 2012

## Effectors Suppress Reactive Oxygen Species Generation

Another vital plant defense response is the generation of reactive oxygen species (ROS) after pathogen recognition, in which plant reduced nicotinamide adenine dinucleotide phosphate oxidase RBOHD plays a crucial role. Botrytis-induced kinase 1 (BIK1) is an RLCK that associates with multiple PRR proteins of plants involved in defense responses. BIK1 interacts with plant PRRs and assembles into a complex that directly phosphorylates RBOHD to trigger ROS generation (Lu et al., 2010; Zhang et al., 2010; Gao et al., 2018). During the plant–pathogen “arms race,” pathogens have evolved effectors to inhibit host immunity by suppressing ROS accumulation in plants. A very recent study of an NIS1 effector of *C. orbiculare* revealed that CoNIS1 can reduce bacterial PAMP flagellin (flg22)- and fungal PAMP chitin-triggered ROS accumulation in *N. benthamiana* by targeting BIK1 and blocking the association between BIK1 and RBOHD (Irieda et al., 2019). Loss of BAK1 or BIK1 impairs *Arabidopsis* immunity to *Colletotrichum* fungi, suggesting that the RLK and RLCK targeted by NIS1 are critical for resistance in the host plant (Irieda et al., 2019). The expression of *CoNIS1* in melon cotyledons induced by an efficient *Agrobacterium* infiltration system markedly suppressed flg22-triggered ROS generation, similar to the observations in *N. benthamiana* (Chen et al., 2021). These results reveal that the ability of NIS1 to suppress ROS burst is conserved in multiple fungus–plant interactions. Other effectors involved in ROS suppression include SIB1 and SIB2 of *C. orbiculare*. Overexpression of *SIB1* increases the susceptibility of *N. benthamiana* to *C. orbiculare*, by suppressing the generation of ROS triggered by both chitin and flg22 (Zhang et al., 2021). These findings indicate that *Colletotrichum* pathogens secrete effector proteins as molecular weapons that modulate ROS accumulation, therefore interfering with host PAMP-triggered immunity.

## Effectors Induce Plant Immunity

Plant pathogens adopt different virulence strategies to infect host cells and, in turn, plants evolve multi-layered immune defenses to recognize pathogen effectors and induce host immunity. During the confrontation between plants and pathogens, plant PRRs can recognize PAMPs and trigger PTI, while plant nucleotide-binding domain leucine-rich repeat containing receptors recognize effectors and induce effector-triggered immunity (Boyd et al., 2013). Plant immune responses include HR induction, ROS production, the activation of defense gene expression, extracellular alkalinization, and callose deposition; together, these mechanisms provide a systemic, durable, and broad spectrum of defense (Prime et al., 2006). It is important to identify PAMPs and effectors serving as resistance inducers to promote sustainable crop protection.

Many PAMPs and effectors from the genus *Colletotrichum* have shown cell death-inducing activities (Table 3). NLPs acting as a class of well-known PAMPs, are conserved in many phytopathogens and strongly induce cell death in eudicot plants. The leucine-rich repeat receptor protein RLP23 forms a constitutive complex with the other RLKs and mediates NLP-triggered immunity by sensing a conserved 20-amino-acid fragment of NLP sequences in *Arabidopsis* (Albert et al., 2015). Six NLP homologs in *C. higginsianum* have necrosis-inducing activities, and they are expressed during the switch to necrotrophy and induce necrotic symptoms in *N. benthamiana* (Kleemann et al., 2012). Furthermore, *C. orbiculare*, which causes anthracnose disease in cucurbit, secretes conserved NLP and NIS effectors. Transient expression of NLP1 or NIS1 also induces cell death in both *N. benthamiana* leaves and melon cotyledons. A mutation that deletes signal peptides and mutations in the heptapeptide motif of NLP1 blocks cell death-inducing activity in *N. benthamiana* but still causes cell death in melon. These results suggest that machinery for NLP1-triggered cell death probably differs among susceptible plants (Chen et al., 2021). Analysis of a series of deletion mutants showed that the carboxy-terminal 32 amino acids of NLP1 are recognized by Cucurbitaceae plants, which is sufficient to trigger cell death in cucumber cotyledons (Azmi et al., 2018).

**TABLE 3:** List of plant immunity-inducing effectors in *Colletotrichum* pathogens.

Effector	*Colletotrichum* pathogen	Expression stage	Biological functions	Sequence conservation	References
ChNLP1	*C. higginsianum*	Biotrophy to necrotrophy switch	Inducing necrosis in *N. benthamiana*	Conserved in fungi and oomycetes	Kleemann et al., 2012
ChCEC3	*C. higginsianum*	Pre-penetration stage and early biotrophic stage	Inducing cell death in *N. benthamiana*, and inducing plant nuclear expansion	Conserved in *Colletotrichum* spp.	Tsushima et al., 2021
NLP1	*C. orbiculare*	Late infection phase	Inducing cell death in *N. benthamiana* and melon	Conserved in fungi and oomycetes	Azmi et al., 2018; Chen et al., 2021
CoNIS1	*C. orbiculare*	Late infection phase	Inducing necrosis in *N. benthamiana*	Conserved in fungi and oomycetes	Yoshino et al., 2012
CEC3	*C. orbiculare*	Pre-penetration stage and early biotrophic stage	Inducing cell death in *N. benthamiana*	Conserved in Colletotrichum spp.	Tsushima et al., 2021
CfCEC3	*C. fructicola*	Pre-penetration stage and early biotrophic stage	Inducing cell death in *N. benthamiana*	Conserved in Colletotrichum spp.	Tsushima et al., 2021
EPL1	*C. falcatum*	Biotrophic phase	Eliciting systemic resistance in sugarcane and HR response in tobacco	Conserved in filamentous fungi	Ashwin et al., 2017
CfPDIP1	*C. falcatum*	Highly expressed between 24 and 72 hpi	Eliciting defense responses in sugarcane and HR response in tobacco	Unknown	Ashwin et al., 2018
CtNUDIX	*C. truncatum*	Late biotrophic phase	Eliciting HR-like cell death in tobacco	Conserved in fungi	Bhadauria et al., 2013

As well as conserved NLPs, many potential PAMPs and proteins that induce HR have also been identified by secretome analysis. For example, *C. falcatum* secretes a cerato-platanin protein called EPL1, which induces HR-like cell death 24 h after infiltration in *N. tabacum* (Ashwin et al., 2017). Another novel protein secreted by *C. falcatum*, CfPDIP1, is also an HR-inducing protein. Functional characterization of distinct domain deletion variants revealed that hydroxyl-deleted variants of CfPDIP1 also rapidly trigger HR (Ashwin et al., 2018). Remarkably, some hemibiotrophic pathogens trigger cell death to signal the transition from biotrophy to necrotrophy. For example, the Nudix hydrolase domain-containing proteins, which are widely distributed among eukaryotes, act as important effectors in phytopathogens by manipulating host defense systems in a hydrolysis activity-dependent manner (Kong et al., 2015; Dong and Wang, 2016). The CtNUDIX effector in *C. truncatum* contains a putative 23-amino acid Nudix hydrolase motif in the carboxy terminus. The full-length protein of CtNUDIX can induce cell death in tobacco, but *Agrobacterium tumefaciens* strains carrying the CtNUDIXΔSP without its signal peptide are unable to induce necrosis phenotypes. Moreover, the recombinant protein from C-terminal enhanced green fluorescent protein fusion to CtNUDIX at the plasma membrane, and precisely overlapped the area of fluorescence for the membrane-selective red fluorescent dye FM4-64. This suggests that the CtNUDIX effector causes cell death-induced activity, which is probably associated with its function in the plasma membrane and extracellular space. However, the plant targets or substrates of CtNUDIX and the mechanism by which CtNUDIX elicits cell death remains to be investigated (Bhadauria et al., 2013). Based on abundant genome resources of *Colletotrichum* spp., recent studies have identified some core effectors conserved within the genus. Comparative genomic analyses revealed core effector of *Colletotrichum* (CEC) proteins, which are conserved in seven *Colletotrichum* species. Taking *C. higginsianum* as an example, ChCEC homologs (*ChCEC2-1*, *ChCEC2-2*, *ChCEC3*, and *ChCEC6*) are highly expressed during infection, and only ChCEC3 can induce cell death in *N. benthamiana*. Notably, homologs of ChCEC3 from *C. orbiculare*, *C. fructicola* and *C. graminicola* can also induce cell death. Also, analysis of subcellular localization shows that plant cells expressing ChCEC3 homologs have greater diameter nucleuses, although the mechanism of this expansion is unknown (Tsushima et al., 2021).

Besides inducing PCD, certain effectors of *Colletotrichum* spp. can also promote host resistance by inducing extracellular alkalinization, ROS production, callose deposition, and the expression of defense-related genes. In *C. falcatum*, the effector EPL1 and its deletion mutant induce acute extracellular alkalinization and H_2_O_2_ accumulation in both the model plant *N. tabacum* and host sugarcane. Relative expression analysis showed that the deletion mutant of *EPL1* significantly promotes the expression of pathogenesis-related genes (Ashwin et al., 2017). Similarly, the effector CfPDIP1 of *C. falcatum* and its deletion mutant also induce a high level of H_2_O_2_ and result in rapid alkalinization in the extracellular space. The expression of important candidate defense-related genes is upregulated in CfPDIP1ΔN1-21-primed canes (Ashwin et al., 2018). Accumulating evidence suggests that some effectors are perceived by plants and act as resistance inducers to activate systemic resistance.

## Concluding Remarks

*Colletotrichum* pathogens pose a serious threat to food security worldwide. Understanding the molecular basis of *Colletotrichum* pathogenesis is very important for developing effective control strategies. In recent years, advances in sequencing technologies and bioinformatics tools have greatly accelerated the identification and evolutionary characterization of a large number of putative effectors in *Colletotrichum* pathogens, and the biological functions of some of these putative effectors have also been investigated. Our understanding of *Colletotrichum* effectors is improving and studies have revealed that these molecules are capable of both suppressing plant defense responses and inducing host resistance. However, our understanding of the plant targets of these effectors and the detailed molecular mechanisms are still in their infancy. Therefore, it is essential to elucidate the modes of action of these effectors and their plant targets, to identify novel modes of resistance against these pathogens. Also, little is known about the translocation routes and signals of fungal effectors. It would be interesting to investigate how *Colletotrichum* effectors are secreted and delivered during pathogen–plant interactions. Such studies would provide novel insights into the molecular mechanisms underlying the virulence functions of *Colletotrichum* effectors, and aid the development of new strategies to combat anthracnose in crops.

## Author Contributions

XL, JM, and DS drafted the manuscript. DS and DD revised the manuscript. All authors read and approved of the manuscript.

## Conflict of Interest

The authors declare that the research was conducted in the absence of any commercial or financial relationships that could be construed as a potential conflict of interest.

## Publisher’s Note

All claims expressed in this article are solely those of the authors and do not necessarily represent those of their affiliated organizations, or those of the publisher, the editors and the reviewers. Any product that may be evaluated in this article, or claim that may be made by its manufacturer, is not guaranteed or endorsed by the publisher.

## References

[B1] Albert I. Bohm H. Albert M. Feiler C. E. Imkampe J. Wallmeroth N. (2015). An RLP23-SOBIR1-BAK1 complex mediates NLP-triggered immunity. *Nat. Plants* 1:15140. 10.1038/nplants.2015.140 27251392

[B2] Alkan N. Friedlander G. Ment D. Prusky D. Fluhr R. (2015). *Simultaneous transcriptome* analysis of *Colletotrichum gloeosporioides* and tomato fruit pathosystem reveals novel fungal pathogenicity and fruit defense strategies. *New Phytol.* 205 801–815. 10.1111/nph.13087 25377514

[B3] Ashwin N. M. R. Barnabas L. Ramesh Sundar A. Malathi P. Viswanathan R. Masi A. (2017). Comparative secretome analysis of *Colletotrichum falcatum* identifies a cerato-platanin protein (EPL1) as a potential pathogen-associated molecular pattern (PAMP) inducing systemic resistance in sugarcane. *J. Proteomics* 169 2–20. 10.1016/j.jprot.2017.05.020 28546091

[B4] Ashwin N. M. R. Barnabas L. Ramesh Sundar A. Malathi P. Viswanathan R. Masi A. (2018). CfPDIP1, a novel secreted protein of *Colletotrichum falcatum*, elicits defense responses in sugarcane and triggers hypersensitive response in tobacco. *Appl. Microbiol. Biotechnol.* 102 6001–6021. 10.1007/s00253-018-9009-2 29728727

[B5] Azmi N. S. A. Singkaravanit-Ogawa S. Ikeda K. Kitakura S. Inoue Y. Narusaka Y. (2018). Inappropriate expression of an NLP effector in *Colletotrichum orbiculare* impairs infection on *Cucurbitaceae cultivars* via plant recognition of the C-terminal region. *Mol. Plant Microbe Int.* 31 101–111. 10.1094/MPMI-04-17-0085-FI 29059009

[B6] Baroncelli R. Amby D. B. Zapparata A. Sarrocco S. Vannacci G. Le Floch G. (2016). Gene family expansions and contractions are associated with host range in plant pathogens of the genus *Colletotrichum*. *BMC Genom.* 17:555. 10.1186/s12864-016-2917-6 27496087 PMC4974774

[B7] Bhadauria V. Banniza S. Vandenberg A. Selvaraj G. Wei Y. (2013). Overexpression of a novel biotrophy-specific *Colletotrichum truncatum* effector, CtNUDIX, in hemibiotrophic fungal phytopathogens causes incompatibility with their host plants. *Eukaryot Cell* 12 2–11. 10.1128/EC.00192-12 22962277 PMC3535838

[B8] Bhadauria V. MacLachlan R. Pozniak C. Cohen-Skalie A. Li L. Halliday J. (2019). Genetic map-guided genome assembly reveals a virulence-governing minichromosome in the lentil anthracnose pathogen *Colletotrichum lentis*. *New Phytol.* 221 431–445. 10.1111/nph.15369 30076781 PMC6668012

[B9] Bhadauria V. Vijayan P. Wei Y. D. Banniza S. (2017). Transcriptome analysis reveals a complex interplay between resistance and effector genes during the compatible lentil-*Colletotrichum lentis* interaction. *Sci. Rep.* 7:42338. 10.1038/srep42338 28186158 PMC5301223

[B10] Boufleur T. R. Massola Junior N. S. Tikami I. Sukno S. A. Thon M. R. Baroncelli R. (2021). Identification and comparison of *Colletotrichum* secreted effector candidates reveal two independent lineages pathogenic to soybean. *Pathogens* 10:520. 10.3390/pathogens10111520 34832675 PMC8625359

[B11] Boyd L. A. Ridout C. O’Sullivan D. M. Leach J. E. Leung H. (2013). Plant-pathogen interactions: disease resistance in modern agriculture. *Trends Genet* 29 233–240. 10.1016/j.tig.2012.10.011 23153595

[B12] Bozkurt T. O. Schornack S. Banfield M. J. Kamoun S. (2012). Oomycetes, effectors, and all that jazz. *Curr. Opin. Plant Biol.* 15 483–492. 10.1016/j.pbi.2012.03.008 22483402

[B13] Buiate E. A. S. Xavier K. V. Moore N. Torres M. F. Farman M. L. Schardl C. L. (2017). A comparative genomic analysis of putative pathogenicity genes in the host-specific sibling species *Colletotrichum graminicola* and *Colletotrichum sublineola*. *BMC Genom.* 18:67. 10.1186/s12864-016-3457-9 28073340 PMC5225507

[B14] Chen J. Inoue Y. Kumakura N. Mise K. Shirasu K. Takano Y. (2021). Comparative transient expression analyses on two conserved effectors of *Colletotrichum orbiculare* reveal their distinct cell death-inducing activities between *Nicotiana benthamiana* and melon. *Mol. Plant Pathol.* 22 1006–1013. 10.1111/mpp.13078 34132478 PMC8295514

[B15] Dallery J. F. Lapalu N. Zampounis A. Pigne S. Luyten I. Amselem J. (2017). Gapless genome assembly of *Colletotrichum higginsianum* reveals chromosome structure and association of transposable elements with secondary metabolite gene clusters. *BMC Genom.* 18:667. 10.1186/s12864-017-4083-x 28851275 PMC5576322

[B16] de Jonge R. van Esse H. P. Kombrink A. Shinya T. Desaki Y. Bours R. (2010). Conserved fungal LysM effector Ecp6 prevents chitin-triggered immunity in plants. *Science* 329 953–955. 10.1126/science.1190859 20724636

[B17] de Queiroz C. B. Correia H. L. N. Santana M. F. Batista D. S. Vidigal P. M. P. Brommonschenkel S. H. (2019). The repertoire of effector candidates in *Colletotrichum lindemuthianum* reveals important information about *Colletotrichum genus* lifestyle. *Appl. Microbiol. Biotechnol.* 103 2295–2309. 10.1007/s00253-019-09639-9 30685810

[B18] Dean R. Van Kan J. A. Pretorius Z. A. Hammond-Kosack K. E. Di Pietro A. Spanu P. D. (2012). The top 10 fungal pathogens in molecular plant pathology. *Mol. Plant Pathol.* 13 414–430. 10.1111/j.1364-3703.2011.00783.x 22471698 PMC6638784

[B19] DeZwaan T. M. Carroll A. M. Valent B. Sweigard J. A. (1999). Magnaporthe grisea pth11p is a novel plasma membrane protein that mediates appressorium differentiation in response to inductive substrate cues. *Plant Cell* 11 2013–2030. 10.1105/tpc.11.10.2013 10521529 PMC144101

[B20] Dodds P. N. Rathjen J. P. (2010). Plant immunity: towards an integrated view of plant-pathogen interactions. *Nat. Rev. Genet.* 11 539–548. 10.1038/nrg2812 20585331

[B21] Dong S. Wang Y. (2016). Nudix effectors: a common weapon in the arsenal of plant pathogens. *PLoS Pathog* 12:e1005704. 10.1371/journal.ppat.1005704 27737001 PMC5063578

[B22] Dou D. Kale S. D. Wang X. Chen Y. Wang Q. Wang X. (2008). Conserved c-terminal motifs required for avirulence and suppression of cell death by *Phytophthora sojae* effector Avr1b. *Plant Cell* 20 1118–1133. 10.1105/tpc.107.057067 18390593 PMC2390733

[B23] Dou D. Zhou J. M. (2012). Phytopathogen effectors subverting host immunity: different foes, similar battleground. *Cell Host Microbe* 12 484–495. 10.1016/j.chom.2012.09.003 23084917

[B24] Gan P. Hiroyama R. Tsushima A. Masuda S. Shibata A. Ueno A. (2021). Telomeres and a repeat-rich chromosome encode effector gene clusters in plant pathogenic *Colletotrichum fungi*. *Environ. Microbiol.* 23 6004–6018. 10.1111/1462-2920.15490 33780109

[B25] Gan P. Ikeda K. Irieda H. Narusaka M. O’Connell R. J. Narusaka Y. (2013). Comparative genomic and transcriptomic analyses reveal the hemibiotrophic stage shift of *Colletotrichum fungi*. *New Phytol.* 197 1236–1249. 10.1111/nph.12085 23252678

[B26] Gao X. Ruan X. Sun Y. Wang X. Feng B. (2018). BAKing up to survive a battle: functional dynamics of BAK1 in plant programmed cell death. *Front. Plant Sci.* 9:1913. 10.3389/fpls.2018.01913 30671069 PMC6331536

[B27] Giraldo M. C. Dagdas Y. F. Gupta Y. K. Mentlak T. A. Yi M. Martinez-Rocha A. L. (2013). Two distinct secretion systems facilitate tissue invasion by the rice blast fungus *Magnaporthe oryzae*. *Nat. Commun.* 4:1996. 10.1038/ncomms2996 23774898 PMC3709508

[B28] Hacquard S. Kracher B. Hiruma K. Munch P. C. Garrido-Oter R. Thon M. R. (2016). Survival trade-offs in plant roots during colonization by closely related beneficial and pathogenic fungi. *Nat. Commun.* 7:11362. 10.1038/ncomms11362 27150427 PMC4859067

[B29] He K. Gou X. Yuan T. Lin H. Asami T. Yoshida S. (2007). BAK1 and BKK1 regulate brassinosteroid-dependent growth and brassinosteroid-independent cell-death pathways. *Curr. Biol.* 17 1109–1115. 10.1016/j.cub.2007.05.036 17600708

[B30] Hsieh D. K. Chuang S. C. Chen C. Y. Chao Y. T. Lu M. J. Lee M. H. (2022). Comparative genomics of three *Colletotrichum scovillei* strains and genetic analysis revealed genes involved in fungal growth and virulence on chili pepper. *Front. Microbiol.* 13:818291. 10.3389/fmicb.2022.818291 35154058 PMC8828978

[B31] Huser A. Takahara H. Schmalenbach W. O’Connell R. (2009). Discovery of pathogenicity genes in the crucifer anthracnose fungus *Colletotrichum higginsianum*, using random insertional mutagenesis. *Mol. Plant Microbe Int.* 22 143–156. 10.1094/MPMI-22-2-0143 19132867

[B32] Irieda H. Inoue Y. Mori M. Yamada K. Oshikawa Y. Saitoh H. (2019). Conserved fungal effector suppresses PAMP-triggered immunity by targeting plant immune kinases. *Proc. Natl. Acad. Sci. U.S.A.* 116 496–505. 10.1073/pnas.1807297116 30584105 PMC6329965

[B33] Irieda H. Maeda H. Akiyama K. Hagiwara A. Saitoh H. Uemura A. (2014). Colletotrichum orbiculare secretes virulence effectors to a biotrophic interface at the primary hyphal neck via exocytosis coupled with SEC22-mediated traffic. *Plant Cell* 26 2265–2281. 10.1105/tpc.113.120600 24850852 PMC4079382

[B34] Isozumi N. Inoue Y. Imamura T. Mori M. Takano Y. Ohki S. (2019). Ca2+-dependent interaction between calmodulin and CoDN3, an effector of *Colletotrichum orbiculare*. *Biochem. Biophys. Res. Commun.* 514 803–808. 10.1016/j.bbrc.2019.05.007 31079920

[B35] Jeong Y. J. Shang Y. Kim B. H. Kim S. Y. Song J. H. Lee J. S. (2010). BAK7 displays unequal genetic redundancy with BAK1 in brassinosteroid signaling and early senescence in *Arabidopsis*. *Mol. Cells* 29 259–266. 10.1007/s10059-010-0024-0 20108170

[B36] Kemmerling B. Schwedt A. Rodriguez P. Mazzotta S. Frank M. Qamar S. A. (2007). The BRI1-associated kinase 1, BAK1, has a brassinolide-independent role in plant cell-death control. *Curr. Biol.* 17 1116–1122. 10.1016/j.cub.2007.05.046 17583510

[B37] Kleemann J. Rincon-Rivera L. J. Takahara H. Neumann U. Ver Loren van Themaat E. van der Does H. C. (2012). Sequential delivery of host-induced virulence effectors by appressoria and intracellular hyphae of the phytopathogen *Colletotrichum higginsianum*. *PLoS Pathog* 8:e1002643. 10.1371/journal.ppat.1002643 22496661 PMC3320591

[B38] Kong G. Zhao Y. Jing M. Huang J. Yang J. Xia Y. (2015). The activation of *Phytophthora effector* Avr3b by plant cyclophilin is required for the nudix hydrolase activity of Avr3b. *PLoS Pathog* 11:e1005139. 10.1371/journal.ppat.1005139 26317500 PMC4552650

[B39] Lacomme C. Santa Cruz S. (1999). Bax-induced cell death in tobacco is similar to the hypersensitive response. *Proc. Natl. Acad. Sci. U.S.A.* 96 7956–7961. 10.1073/pnas.96.14.7956 10393929 PMC22169

[B40] Lelwala R. V. Korhonen P. K. Young N. D. Scott J. B. Ades P. K. Gasser R. B. (2019). Comparative genome analysis indicates high evolutionary potential of pathogenicity genes in *Colletotrichum tanaceti*. *PLoS One* 14:e0212248. 10.1371/journal.pone.0212248 31150449 PMC6544218

[B41] Liang X. F. Shang S. P. Dong Q. Y. Wang B. Zhang R. Gleason M. L. (2018). Transcriptomic analysis reveals candidate genes regulating development and host interactions of *Colletotrichum fructicola*. *BMC Genom.* 19:557. 10.1186/s12864-018-4934-0 30055574 PMC6064131

[B42] Lievens B. Houterman P. M. Rep M. (2009). Effector gene screening allows unambiguous identification of *Fusarium oxysporum* f. sp. lycopersici races and discrimination from other formae speciales. *FEMS Microbiol. Lett.* 300 201–215. 10.1111/j.1574-6968.2009.01783.x 19799634

[B43] Lu D. Wu S. Gao X. Zhang Y. Shan L. He P. (2010). A receptor-like cytoplasmic kinase, BIK1, associates with a flagellin receptor complex to initiate plant innate immunity. *Proc. Natl. Acad. Sci. U.S.A.* 107 496–501. 10.1073/pnas.0909705107 20018686 PMC2806711

[B44] Miya A. Albert P. Shinya T. Desaki Y. Ichimura K. Shirasu K. (2007). CERK1, a LysM receptor kinase, is essential for chitin elicitor signaling in *Arabidopsis*. *Proc. Natl. Acad. Sci. U.S.A.* 104 19613–19618. 10.1073/pnas.0705147104 18042724 PMC2148337

[B45] Monaghan J. Zipfel C. (2012). Plant pattern recognition receptor complexes at the plasma membrane. *Curr. Opin. Plant Biol.* 15 349–357. 10.1016/j.pbi.2012.05.006 22705024

[B46] Mosquera G. Giraldo M. C. Khang C. H. Coughlan S. Valent B. (2009). Interaction transcriptome analysis identifies *Magnaporthe oryzae* BAS1-4 as biotrophy-associated secreted proteins in rice blast disease. *Plant Cell* 21 1273–1290. 10.1105/tpc.107.055228 19357089 PMC2685627

[B47] O’Connell R. Herbert C. Sreenivasaprasad S. Khatib M. Esquerre-Tugaye M. T. Dumas B. (2004). A novel arabidopsis-colletotrichum pathosystem for the molecular dissection of plant-fungal interactions. *Mol. Plant Microbe Int.* 17 272–282. 10.1094/MPMI.2004.17.3.272 15000394

[B48] O’Connell R. J. Thon M. R. Hacquard S. Amyotte S. G. Kleemann J. Torres M. F. (2012). Lifestyle transitions in plant pathogenic *Colletotrichum fungi* deciphered by genome and transcriptome analyses. *Nat. Genet.* 44 1060–1065. 10.1038/ng.2372 22885923 PMC9754331

[B49] Pemberton C. L. Salmond G. P. (2004). The Nep1-like proteins-a growing family of microbial elicitors of plant necrosis. *Mol. Plant Pathol.* 5 353–359. 10.1111/j.1364-3703.2004.00235.x 20565603

[B50] Perfect S. E. Hughes H. B. O’Connell R. J. Green J. R. (1999). Colletotrichum: a model genus for studies on pathology and fungal-plant interactions. *Fungal Genet Biol.* 27 186–198. 10.1006/fgbi.1999.1143 10441444

[B51] Petre B. Saunders D. G. Sklenar J. Lorrain C. Win J. Duplessis S. (2015). Candidate effector proteins of the rust pathogen melampsora larici-populina target diverse plant cell compartments. *Mol. Plant Microbe Int.* 28 689–700. 10.1094/MPMI-01-15-0003-R 25650830

[B52] Plaumann P. L. Schmidpeter J. Dahl M. Taher L. Koch C. (2018). A dispensable chromosome is required for virulence in the hemibiotrophic plant pathogen *Colletotrichum higginsianum*. *Front. Microbiol.* 9:1005. 10.3389/fmicb.2018.01005 29867895 PMC5968395

[B53] Plissonneau C. Benevenuto J. Mohd-Assaad N. Fouche S. Hartmann F. E. Croll D. (2017). Using population and comparative genomics to understand the genetic basis of effector-driven fungal pathogen evolution. *Front. Plant Sci.* 8:119. 10.3389/fpls.2017.00119 28217138 PMC5289978

[B54] Prime A. P. G. Conrath U. Beckers G. J. Flors V. Garcia-Agustin P. Jakab G. (2006). Priming: getting ready for battle. *Mol. Plant Microbe Int.* 19 1062–1071. 10.1094/MPMI-19-1062 17022170

[B55] Raffaele S. Kamoun S. (2012). Genome evolution in filamentous plant pathogens: why bigger can be better. *Nat. Rev. Microbiol.* 10 417–430. 10.1038/nrmicro2790 22565130

[B56] Rafiqi M. Ellis J. G. Ludowici V. A. Hardham A. R. Dodds P. N. (2012). Challenges and progress towards understanding the role of effectors in plant-fungal interactions. *Curr. Opin. Plant Biol.* 15 477–482. 10.1016/j.pbi.2012.05.003 22658704

[B57] Rao S. Nandineni M. R. (2017). Genome sequencing and comparative genomics reveal a repertoire of putative pathogenicity genes in chilli anthracnose fungus *Colletotrichum truncatum*. *PLoS One* 12:e0183567. 10.1371/journal.pone.0183567 28846714 PMC5573122

[B58] Rao S. Sharda S. Oddi V. Nandineni M. R. (2018). The landscape of repetitive elements in the eefined genome of chilli anthracnose fungus *Colletotrichum truncatum*. *Front. Microbiol.* 9:2367. 10.3389/fmicb.2018.02367 30337918 PMC6180176

[B59] Rech G. E. Sanz-Martin J. M. Anisimova M. Sukno S. A. Thon M. R. (2014). Natural selection on coding and noncoding DNA sequences is associated with virulence genes in a plant pathogenic fungus. *Genome Biol. Evol.* 6 2368–2379. 10.1093/gbe/evu192 25193312 PMC4202328

[B60] Robin G. P. Kleemann J. Neumann U. Cabre L. Dallery J. F. Lapalu N. (2018). Subcellular localization screening of *Colletotrichum higginsianum* effector candidates identifies fungal proteins targeted to plant peroxisomes, golgi bodies, and microtubules. *Front. Plant Sci.* 9:562. 10.3389/fpls.2018.00562 29770142 PMC5942036

[B61] Saitoh H. Fujisawa S. Mitsuoka C. Ito A. Hirabuchi A. Ikeda K. (2012). Large-scale gene disruption in *Magnaporthe oryzae* identifies MC69, a secreted protein required for infection by monocot and dicot fungal pathogens. *PLoS Pathog* 8:e1002711. 10.1371/journal.ppat.1002711 22589729 PMC3349759

[B62] Sanz-Martin J. M. Pacheco-Arjona J. R. Bello-Rico V. Vargas W. A. Monod M. Diaz-Minguez J. M. (2016). A highly conserved metalloprotease effector enhances virulence in the maize anthracnose fungus *Colletotrichum graminicola*. *Mol. Plant Pathol.* 17 1048–1062. 10.1111/mpp.12347 26619206 PMC6638349

[B63] Seidl M. F. Van den Ackerveken G. (2019). Activity and phylogenetics of the broadly occurring family of microbial Nep1-like proteins. *Ann. Rev. Phytopathol.* 57 367–386. 10.1146/annurev-phyto-082718-100054 31283435

[B64] Shang S. Wang B. Zhang S. Liu G. Liang X. Zhang R. (2020). A novel effector CfEC92 of *Colletotrichum fructicola* contributes to glomerella leaf spot virulence by suppressing plant defences at the early infection phase. *Mol. Plant Pathol.* 21 936–950. 10.1111/mpp.12940 32512647 PMC7279981

[B65] Stephenson S. A. Hatfield J. Rusu A. G. Maclean D. J. Manners J. M. (2000). CgDN3: an essential pathogenicity gene of *Colletotrichum gloeosporioides* necessary to avert a hypersensitive-like response in the host *Stylosanthes guianensis*. *Mol. Plant Microbe Int.* 13 929–941. 10.1094/MPMI.2000.13.9.929 10975650

[B66] Takahara H. Hacquard S. Kombrink A. Hughes H. B. Halder V. Robin G. P. (2016). *Colletotrichum higginsianum* extracellular LysM proteins play dual roles in appressorial function and suppression of chitin-triggered plant immunity. *New Phytol.* 211 1323–1337. 10.1111/nph.13994 27174033

[B67] Tanaka K. Nguyen C. T. Liang Y. Cao Y. Stacey G. (2013). Role of LysM receptors in chitin-triggered plant innate immunity. *Plant Signal Behav.* 8:e22598. 10.4161/psb.22598 23221760 PMC3745565

[B68] Tsushima A. Gan P. Kumakura N. Narusaka M. Takano Y. Narusaka Y. (2019). Genomic plasticity mediated by transposable elements in the plant pathogenic fungus *Colletotrichum higginsianum*. *Genome Biol. Evol.* 11 1487–1500. 10.1093/gbe/evz087 31028389 PMC6535813

[B69] Tsushima A. Narusaka M. Gan P. Kumakura N. Hiroyama R. Kato N. (2021). The conserved *Colletotrichum* spp. effector candidate CEC3 induces nuclear expansion and cell death in plants. *Front. Microbiol.* 12:682155. 10.3389/fmicb.2021.682155 34539598 PMC8446390

[B70] van Esse H. P. Bolton M. D. Stergiopoulos I. de Wit P. J. Thomma B. P. (2007). The chitin-binding *Cladosporium fulvum* effector protein Avr4 is a virulence factor. *Mol. Plant Microbe Int.* 20 1092–1101. 10.1094/MPMI-20-9-1092 17849712

[B71] Wang Y. Wang Y. (2018). Trick or treat: microbial pathogens evolved apoplastic effectors modulating plant susceptibility to infection. *Mol. Plant Microbe Int.* 31 6–12. 10.1094/MPMI-07-17-0177-FI 29090656

[B72] Yoshino K. Irieda H. Sugimoto F. Yoshioka H. Okuno T. Takano Y. (2012). Cell death of nicotiana benthamiana is induced by secreted protein NIS1 of *Colletotrichum orbiculare* and is suppressed by a homologue of CgDN3. *Mol. Plant Microbe Int.* 25 625–636. 10.1094/MPMI-12-11-0316 22352720

[B73] Zhang J. Li W. Xiang T. Liu Z. Laluk K. Ding X. (2010). Receptor-like cytoplasmic kinases integrate signaling from multiple plant immune receptors and are targeted by a *Pseudomonas* syringae effector. *Cell Host Microbe* 7 290–301. 10.1016/j.chom.2010.03.007 20413097

[B74] Zhang L. Huang X. He C. Zhang Q. Y. Zou X. Duan K. (2018). Novel fungal pathogenicity and leaf defense strategies are revealed by simultaneous transcriptome analysis of *Colletotrichum fructicola* and strawberry infected by this fungus. *Front. Plant Sci.* 9:434. 10.3389/fpls.2018.00434 29922301 PMC5996897

[B75] Zhang R. Isozumi N. Mori M. Okuta R. Singkaravanit-Ogawa S. Imamura T. (2021). Fungal effector SIB1 of *Colletotrichum orbiculare* has unique structural features and can suppress plant immunity in *Nicotiana benthamiana*. *J. Biol. Chem.* 297:101370. 10.1016/j.jbc.2021.101370 34756891 PMC8633582

